# Phage PM16 Therapy Induce Long-Term Protective Immunity Against *Proteus mirabilis* via Macrophage Priming

**DOI:** 10.3390/pathogens15010099

**Published:** 2026-01-16

**Authors:** Lina Al Allaf, Anton V. Chechushkov, Vera V. Morozova, Yulia N. Kozlova, Tatiana A. Ushakova, Nina V. Tikunova

**Affiliations:** Laboratory of Molecular Microbiology, Institute of Chemical Biology and Fundamental Medicine, Siberian Branch of Russian Academy of Sciences, Novosibirsk 630090, Russia; allaflina@1bio.ru (L.A.A.); morozova@1bio.ru (V.V.M.); yulia1979@1bio.ru (Y.N.K.); ushakova@1bio.ru (T.A.U.)

**Keywords:** phage therapy, *Proteus mirabilis*, humoral immunity, PM16, bacteriophage, macrophage, cytokines

## Abstract

Bacteriophages, traditionally viewed solely as antibacterial agents, are increasingly being studied for their immunomodulatory properties. In this study, we demonstrate that PM16 phage therapy not only effectively controls subcutaneous *Proteus mirabilis* infection in mice but also induces long-term specific humoral immunity against subsequent reinfection. This immunomodulatory effect was dose-dependent. In vitro, PM16 directly activates macrophages, leading to increased production of proinflammatory cytokines (tumor necrosis factor-α and interleukin-1β) and inducible nitric oxide synthase, and enhances macrophage bactericidal activity against *P. mirabilis*. We assume that the enhancement of the adaptive immune response is mediated not by the phage acting as a classical antigenic adjuvant but by its ability to prime innate immune cells, specifically macrophages. This priming leads to more efficient bacterial clearance, antigen presentation, and the formation of protective immunological memory.

## 1. Introduction

*Proteus mirabilis* is a Gram-negative, motile, non-sporulating bacterium from the *Enterobacteriaceae* family. This bacterium is a pathogen of significant clinical concern, particularly as a leading cause of catheter-associated and complicated urinary tract infections (UTIs), where it is implicated in 10–44% of long-term catheter-associated cases [1]. Its virulence is enhanced by swarming motility, robust biofilm formation, and urease production, which can lead to severe complications such as urolithiasis, bacteremia, and sepsis [1,2]. Importantly, the therapeutic management of *P. mirabilis* is increasingly compromised by a high prevalence of antibiotic resistance [3], with recent studies reporting multi-drug resistance in up to 48% of isolates and the emergence of pan-drug-resistant strains [4]. Control of UTIs caused by antibiotic-sensitive *Proteus* strains is difficult due to the “shield” provided by the biofilm formation [5]. The *Proteus* features, including its escalating resistance, underscores the urgent need for alternative therapeutic strategies, among which phage therapy has re-emerged as a promising precision antimicrobial approach [6].

Bacteriophages (phages) are traditionally regarded as highly specific antimicrobial agents that lyse their bacterial hosts. However, the regulatory frameworks, historically designed for stable chemical antibiotics, must adapt to accommodate phages as evolving biological entities, particularly concerning manufacturing standards, clinical trial design for narrow-spectrum agents, and pathways for updating therapies against resistant strains [7,8]. Clinical efficacy can be limited by narrow host ranges, the potential for bacterial resistance, and complex unpredictable interactions with the host immune system [9]. In turn, a complex interplay between the particular phage, the infecting bacterium, and the mammalian host’s immune system can enhance the efficacy of phage therapy [10]. This interaction can lead to a beneficial “phage-immune synergy”, wherein bacterial clearance is achieved through a combination of direct phage-mediated lysis and the recruitment of host immune components [11]. Notably, while some phages exhibit immunomodulatory properties, clinical trials using phages as standalone antibacterial agents have sometimes failed to demonstrate clear superiority over standard care, highlighting a gap in our understanding [12]. Therefore, a pivotal and unresolved question is whether phages function merely as direct lytic agents or can also act as strategic immunomodulators that actively prime and shape pathogen-specific adaptive immunity. Investigating these phage–immune interactions—specifically the potential for phages to serve as immune adjuvants—is essential for optimizing therapeutic protocols, overcoming current limitations, and achieving durable clinical outcomes against resilient pathogens like *P. mirabilis* [13,14].

It has been shown that phages interact with the mammalian immune system in several ways [15], including phage phagocytosis [16], the recognition of phage nucleic acids by intracellular pattern recognition receptors (PRRs) such as Toll-like receptor 9 (TLR9), the production of anti-phage antibodies, and the modulation of cytokine responses [17]. Despite this, the mechanisms by which phages influence the development of adaptive immunity against bacterial pathogens remain poorly understood.

Our previous study demonstrated that intravenous administration of the *P. mirabilis* phage PM16 into mice induces a transient increase in blood levels of the tumor necrosis factor-α (TNF-α), interferon-γ (IFN-γ), and interleukins (IL-1β and IL-6) [18]. However, it fails to elicit a long-term anti-phage antibody response, suggesting an inability of the particular phage to independently induce B-cell memory, possibly due to suppressed IL-27 [18]. Therefore, it was hypothesized that the PM16 phage could function as an adjuvant during *P. mirabilis* infection, enhancing the specific adaptive immune response against the pathogen.

The aim of the current study is to evaluate the effect of PM16 therapy on the course of the first and second *P. mirabilis* infection in vivo, to investigate the development of a pathogen-specific antibody response, and to elucidate the underlying mechanism by studying the direct interaction between PM16 and macrophages in vitro including the production of proinflammatory cytokines and inducible nitric oxide synthase (iNOS), as well as macrophage bactericidal activity.

## 2. Materials and Methods

### 2.1. Animals

A cohort of two-month-old male Balb/c mice was procured from the animal care facility at the State Research Center of Virology and Biotechnology VECTOR in Novosibirsk. Mice were housed under controlled light–dark conditions and provided with food and water ad libitum. All procedures performed on animals were in accordance with the ethical standards of EU Directive 2010/63/EU and received approval from the Inter-institutional Bioethics Committee of the Institute of Cytology and Genetics SB RAS, Russia (protocol code 70, date of approval 21 January 2021).

### 2.2. P. mirabilis and Its Podophage PM16

The *P. mirabilis* CEMTC 73 strain and its podophage PM16 [19] were sourced from the Collection of Extremophilic Microorganisms and Type Cultures (CEMTC) at the Institute of Chemical Biology and Fundamental Medicine (ICBFM), SB RAS, and the bacterial strain was cultivated in Lysogeny Broth (LB) at 37 °C. Previously, the susceptibility of the *P. mirabilis* CEMTC 73 strain to antibiotics was tested using the disk diffusion method (OXOID, Basingstoke, UK) in accordance with the EUCAST recommendations (https://www.eucast.org, accessed on 25 June 2025). The following antimicrobials were used: beta-lactams, aminoglycosides, fluoroquinolones, chloramphenicol, and sulphonamides, and it was indicated that this strain is resistant to cefotaxime and levofloxacin.

To propagate the PM16 podophage, *P. mirabilis* CEMTC 73 culture was grown to OD600 of 0.6, inoculated with PM16 at a multiplicity of infection (MOI) of 0.1, and incubated at 37 °C with agitation until the onset of bacterial lysis. Phage particles were precipitated from the lysate using polyethylene glycol 8000 (Appli-Chem, Darmstadt, Germany) in the presence of 2.5 M sodium chloride. Following centrifugation, the resulting pellet was resuspended in phosphate-buffered saline (PBS), pH 8.0. Phage purification was carried out by centrifugation (22,000 rpm, 2 h, and 4 °C) through a cesium chloride gradient [20]. After dialysis against PBS, the phage titer was measured as 5 × 10^11^ plaque-forming units per milliliter (PFU/mL).

### 2.3. Limulus Amebocyte Lysate Assay

To assess endotoxin content, purified PM16 was serially diluted in sterile 0.9% NaCl to final concentrations ranging from 10^6^ to 10^12^ PFU/mL. The endotoxin levels of these dilutions were quantified using the limulus amebocyte lysate (LAL) assay (Charles River Laboratories Inc., Charleston, SC, USA) in accordance with the manufacturer’s protocol. For parenteral pharmaceutical formulations, an endotoxin concentration of 0.5 endotoxin units (EU) per milliliter is considered acceptable. A PM16 preparation with a titer of 10^9^ PFU/mL was found to correspond to an endotoxin level of 0.5 EU/mL.

### 2.4. Subcutaneous Infection Model

Balb/c mice were infected with *P. mirabilis* using 10^8^ CFU in 100 µL of PBS per mouse. One day later, the mice were divided into 4 groups (*n* = 8) based on our previous observations on infiltrate sizes. The groups were conditioned in separate ventilated units of AWTech Vent-Biom 1, a ventilated animal conditioning system used to prevent cross-contamination. The only inclusion criteria were the strain (Balb/c) and age (1.5 months); no exclusion criteria were applied, as the study design was observational. Each mouse was treated with different concentrations of PM16 (all in 100 µL of PBS): the first group received only PBS; the second, third, and fourth groups were injected with 10^7^ PFU per mouse, 10^8^ PFU per mouse, and 10^9^ PFU per mouse, respectively. All treatments were applied laterally to the site where *P. mirabilis* was injected. Four weeks after the first *P. mirabilis* infection, the second *P. mirabilis* infection procedure was performed (10^8^ CFU in 100 µL of PBS) but without applying the phage therapy. The infiltrate size was monitored on days 3 and 10 after the onset of both the first and second infection. The infiltrate sizes were estimated using the caliper to measure two orthogonal dimensions of infiltrate with the subsequent calculation of the geometric mean of both using the formula diameter = SQRT (L × W), where L is the one dimension (arbitrary—length) and W is another dimension (arbitrary—width). Blood samples were collected two and four weeks after the first infection. Individual serum samples from each mouse were used for immunological experiments.

### 2.5. Statistics

All data are presented as mean ± SD. For inflammatory infiltrate size at each time point (days 3 and 10 of the first and second infections), the Brown Forsythe and Welch ANOVA test was used, followed by Games–Howell’s multiple comparisons test to com-pare each phage-treated group to the control group (0 PFU). A *p*-value of less than 0.05 was considered statistically significant.

### 2.6. Assessment of Serum IgG to P. mirabilis Infection

*P. mirabilis* cells were immobilized on the surface of 96-well mu-plates (ibidi, Gräfelfing, Germany) and opsonized using the collected mice sera (dilution 1:500) overnight at 4 °C. Then, wells were washed three times with PBS and stained using anti-IgG Alexa Fluor 488-conjugated donkey anti-mouse antibodies (Invitrogen, Waltham, MA, USA). Bacterial cells were stained using intercalating stain Hoechst 33342 (Life technologies, Carlsbad, CA, USA). Fluorescent signals were detected using LSM710 confocal microscope at 1000× magnification. Images were acquired using a 405 nm laser line for Hoechst 33342 (detection window: 415–480 nm) and a 488 nm laser line for Alexa Fluor 488 (detection window: 493–580 nm). Sequential scanning was used to eliminate crosstalk between channels. All images were acquired in a single session using identical microscope settings to ensure valid comparison between subgroups. Image segmentation and measurement were performed with the CellProfiler 3.0 software. Object identification was first conducted based on the anti-IgG signal in the AF488 channel to demarcate bacterial cells. Subsequently, segmentation based on the Hoechst signal was performed, applying a threshold adjustment of +0.5 µm to the nuclear stain to ensure the resulting masks encompassed the periplasmic space. This established segmentation protocol was then uniformly applied to all subsequent images from the remaining serum samples. The mean fluorescence intensity (MFI) per identified bacterial cell was calculated.

### 2.7. Preparation of Stimulated Macrophages

Primary murine bone marrow-derived macrophages were plated at 5000 cells per glass-bottom dish or 24-well plates and stimulated with 20 ng/mL Granulocyte–Macrophage Colony-Stimulating Factor (GM-CSF) for 24 h in DMEM/F12 culture medium supplemented with Fetal Calf Serum (10%) at 37 °C.

### 2.8. Real-Time PCR

To characterize the macrophage-intrinsic response to PM16, cytokine and activation marker expressions were measured in primary murine bone marrow-derived macro-phages stimulated with GM-CSF and exposed to purified PM16 at a different titer in the absence of bacteria. Total RNA was isolated 6, 24, and 48 h after phage addition. cDNA was synthesized using standard reverse transcription procedures, and transcript levels of proinflammatory (IL-1β, TNFα, and iNOS), regulatory (IL-10), and polarization-associated (Arg1, IL-12p40, and IL-23p19) genes were quantified by real-time PCR using gene-specific intron-skipping primers (Table 1) designed with NCBI Primer-BLAST online tool (https://www.ncbi.nlm.nih.gov/tools/primer-blast/; accessed on 5 January 2026) so that each primer should cover the exon–exon junction on target mRNA. The amplification was performed using BioMaster RT-qPCR SYBR Blue (Biolabmix LLC, Novosibirsk, Russia) and the real-time machine CFX96 (Biorad, Hercules, CA, USA). The conditions for PCR were as follows: initial denaturation: 95 °C (5 min); cycling: 95 °C for 15 s; annealing: Tm + 3 °C for 15 s; and elongation: 72 °C for 30 s. The maximum number of 34 cycles was utilized for each primer set. Relative expression was calculated using the ΔΔCt method, with GM-CSF-stimulated macrophages without PM16 serving as the reference control. Gene expression values were normalized to β-actin (Actb) as the housekeeping gene. All data were presented as mean ± SD. For cytokine production level at each time point, the Welch’s *t* test was used. A *p*-value of less than 0.05 was considered significant.

### 2.9. Microscopy and Bacterial Enumeration

To determine whether PM16 enhances macrophage capacity for bacterial uptake and intracellular killing, GM-CSF-stimulated primary murine bone marrow-derived macrophages were then incubated with purified PM16 for 30 min at different titers (0, 10^4^ PFU/mL, 10^5^ PFU/mL, 10^6^ PFU/mL, or 10^7^ PFU/mL). Hoechst-labeled *P. mirabilis* was added at 10^7^ CFU/well in the presence or absence of normal mouse serum obtained from non-immune BALB/c mice (the serum has been characterized as non-binding for *P. mirabilis* and PM16 with ELISA and microscopy experiments). Macrophages were imaged 5 h after bacterial addition using confocal microscopy. Intracellular bacteria were enumerated using CellProfiler software, and the median number of bacteria per macrophage was calculated. Three independent experiments were performed, yielding three biological replicates per group. All data are presented as mean ± SD. For bacterial enumeration at each PM16 concentration, the two-way ANOVA test was used, followed by Sidak’s multiple comparisons test. A *p*-value of less than 0.05 was considered statistically significant. This approach enabled quantitative assessment of phage-induced enhancement of bacterial internalization.

### 2.10. Measurement of Nitric Oxide Production

To assess nitric oxide (NO) production in response to PM16 exposure, GM-CSF-stimulated primary murine bone marrow-derived macrophages were subsequently incubated with purified PM16 for 5 h at titers of 0, 10^4^ PFU/mL, 10^5^ PFU/mL, 10^6^ PFU/mL, or 10^7^ PFU/mL). Intracellular NO production was quantified by adding the fluorescent probe 2′,7′-dichlorofluorescin diacetate (DCF-DA) to the cultures, followed by measurement of fluorescence intensity as an indicator of reactive nitrogen species. Fluorescence values from GM-CSF-only controls were used as the baseline for comparison. Two-way ANOVA test was used to estimate NO production by macrophages, followed by Sidak’s multiple comparisons test. A *p*-value of less than 0.05 was considered statistically significant. This assay enabled evaluation of PM16-induced enhancement of macrophage NO production.

### 2.11. Analysis of Bacterial Survival Within Macrophages

To quantify the impact of PM16 on intracellular *P. mirabilis* survival, GM-CSF-stimulated primary murine bone marrow-derived macrophages were seeded into 24-well plates, stimulated with GM-CSF for 24 h, and then exposed to *P. mirabilis* (10^7^ CFU/well) for 5 h. After the loading period, cultures were treated with PM16 at the indicated titers (0, 10^4^ PFU/mL, 10^5^ PFU/mL, 10^6^ PFU/mL, or 10^7^ PFU/mL) with or without the addition of mouse serum. Twenty-four h after phage addition, macrophages were lysed with sterile water to release intracellular bacteria. Lysates were serially diluted (1:100; 1:1000; 1:10,000; and 1:100,000) and plated onto non-selective agar plates to enumerate viable *P. mirabilis* colonies. Plates were incubated overnight at 37 °C and CFUs per dilution were counted; confluent growth was recorded as >100 colonies. The number of colonies recovered from macrophage lysates was used to assess PM16-dependent, dose-responsive effects on intracellular bacterial viability. Data were analyzed using two-way ANOVA, followed by Sidak’s multiple comparisons test.

## 3. Results

### 3.1. PM16 Phage Therapy Attenuates P. mirabilis Infection and Induces Long-Term Protective Immunity

**Figure 1:**
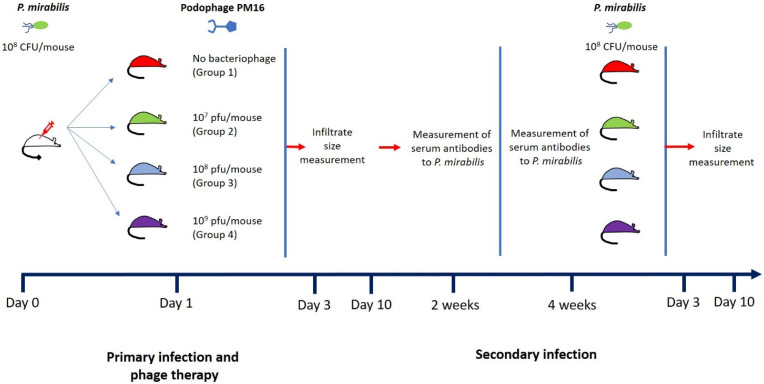
Subcutaneous *P. mirabilis* infection model. Mice were infected with 10^8^ CFU of *P. mirabilis* and, after 24 h, received a single dose of PM16 at varying titers (0, 10^7^ PFU/mouse, 10^8^ PFU/mouse, or 10^9^ PFU/mouse) lateral to the infection site. The infiltrate sizes were measured on days 3 and 10 after injection. Two and four weeks after the first injection, blood serum samples were collected under isoflurane sedation. Four weeks after the first injection, mice were again infected with 10^8^ CFU of *P. mirabilis*, and the infiltrate sizes were measured again at days 3 and 10.

To evaluate the therapeutic and immunomodulatory efficacy of the PM16 phage in vivo, we established a murine model of subcutaneous *P. mirabilis* infection. Mice were infected with 10^8^ CFU of *P. mirabilis* and, after 24 h, received a single dose of PM16 at varying titers (0, 10^7^ PFU/mouse, 10^8^ PFU/mouse, or 10^9^ PFU/mouse) lateral to the infection site (Figure 1). The primary outcome measure was the size of the local inflammatory infiltrate.

**Figure 2:**
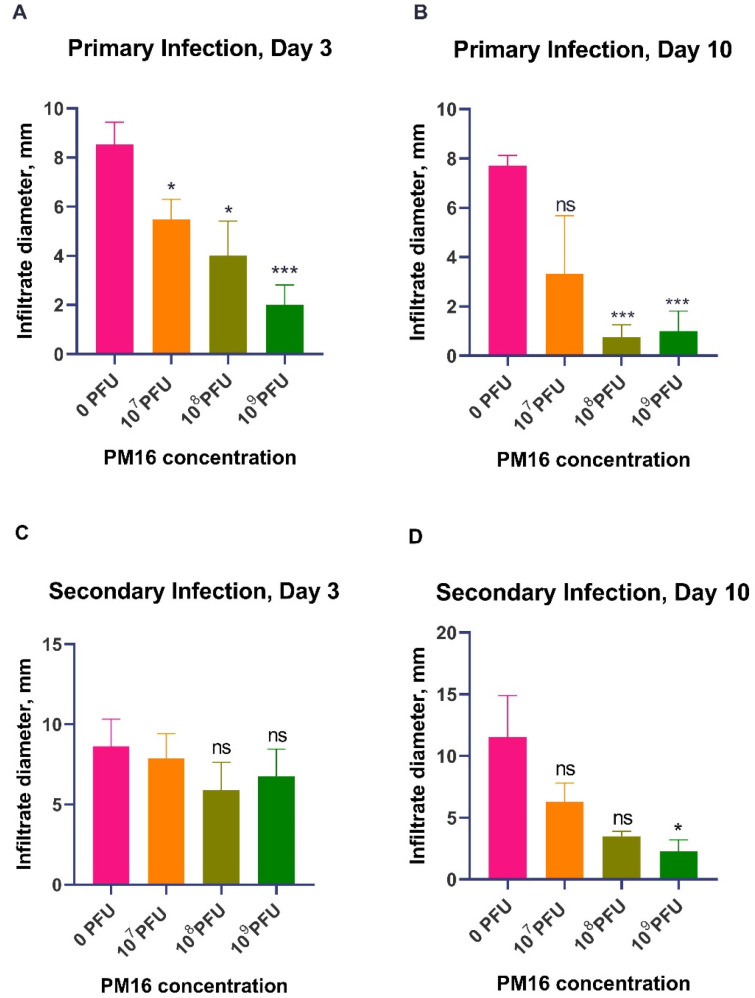
Persistence PM16 phage therapy attenuates primary infection and confers protective immunity against *P. mirabilis* reinfection. (**A**,**B**) Primary infection. Mice (n = 8 per group) were infected subcutaneously with 10^8^ CFU of *P. mirabilis* and treated 24 h later with a single dose of PM16 (0, 10^7^ PFU, 10^8^ PFU, or 10^9^ PFU). Inflammatory infiltrate size was measured on day 3 (**A**) and day 10 (**B**) post-infection. Data are presented as mean ± SEM. Significance was determined by Welch’s ANOVA with Games–Howell post hoc test comparing each group to the control (0 PFU). * *p* < 0.05, *** *p* < 0.001, and “ns” for non-significant. (**C**,**D**) The second infection challenge. The same cohorts of mice were rechallenged with *P. mirabilis* at the contralateral site four weeks after the primary infection without phage therapy. Infiltrate size was measured on day 3 (**C**) and day 10 (**D**) post-reinfection. Data are presented as mean ± SEM. Statistical analysis is the same as in (**A**,**B**).

PM16 therapy exhibited a significant dose-dependent reduction in infiltrate size during *P. mirabilis* infection (Figure 2A). By day 3 post-infection, all phage-treated groups had notably smaller infiltrates compared to the control group (0 PFU). The control group developed infiltrates with a mean size of 8.5 mm, while mice treated with 10^9^ PFU of PM16 exhibited a mean infiltrate size of 2.0 mm (*p* = 0.0002). This therapeutic effect became more pronounced by day 10 (Figure 2B). While the control group still presented significant infiltrates (*mean* = 7.7 mm), the protective effect was the highest in the 10^8^ PFU and 10^9^ PFU groups (*mean* = ~1.0 mm, *p* ≤ 0.0002), with several mice showing complete resolution. In contrast, the lowest dose (10^7^ PFU) was no longer different from the control by day 10 (*p* = 0.094), underscoring the dose-dependent nature of the response.

To determine residual immunity after the bacterial clearance, we challenged the same mice with the second *P. mirabilis* infection four weeks later, without phage therapy (Figure 1). The measurement of the size of the local inflammatory infiltrate upon reinfection indicated that a single prior treatment with PM16 induced robust, dose-dependent protective immunity (Figure 2C,D). Mice initially treated with high-dose PM16 (10^8^ and 10^9^ PFU) were profoundly protected. On day 3, no significant difference was observed in infiltrate sizes. The control group and mice that were injected with 10^7^ PFU had mean infiltrate sizes of 8.6 and 7.8 mm, while groups that were injected with 10^8^ and 10^9^ PFU during the first infection had slightly lower (though not significantly) mean infiltrate sizes of 5.9 and 6.8 mm. However, by day 10, we observed the pattern of decreased infiltrate sizes in accordance with the increased bacteriophage dose during the first injection, with a mean infiltrate size for Group 3 and Group 4 of 3.5 mm (*p* < 0.001) and 2.25 mm (*p* = 0.002). In stark contrast, the control group developed infiltrates of a similar magnitude during both infections (mean = 11 mm). The lowest dose group (10^7^ PFU) also showed a decreased but not statistically significant mean infiltrate size of 6.3 mm compared to the control (Figure 2C,D).

### 3.2. PM16 Phage Therapy Potentiates a Robust and Sustained Humoral Immune Response Against P. mirabilis

**Figure 3:**
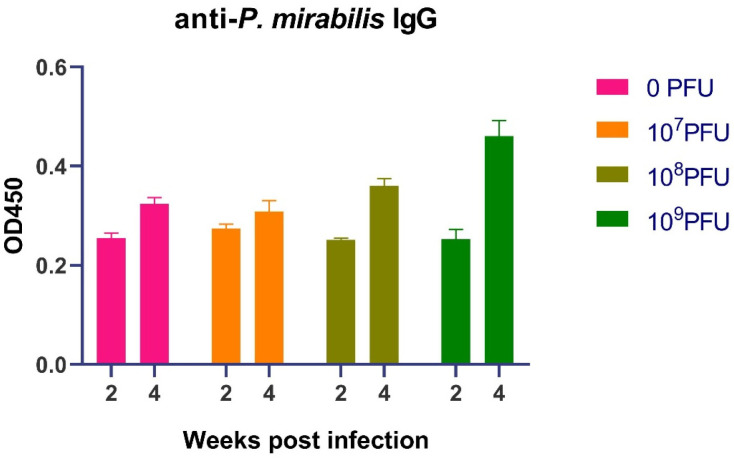
PM16 phage therapy potentiates a robust and sustained humoral immune response against *P. mirabilis*. Serum levels of *P. mirabilis*-specific IgG were measured by ELISA at two and four weeks post-primary infection in mice (n = 8 per group) treated with the indicated doses of PM16. At two weeks, all groups showed equivalent, low-level IgG titers, confirming a consistent initial antigenic stimulus. By four weeks, a significant dose-dependent enhancement of the antibody response was observed in the high-dose phage groups. Data are presented as individual data points with mean ± SEM.

The protection against reinfection suggested the induction of adaptive immunological memory. To investigate the role of humoral immunity, we quantified serum levels of *P. mirabilis*-specific IgG following the first infection. The initial humoral response, measured at two weeks post-infection, was uniform across all groups (Figure 3). IgG levels in all cohorts clustered within a narrow range (approximately 0.225 to 0.285 OD), indicating a nascent, low-titer antibody response. This early equivalence confirms that the initial antigenic stimulus was consistent across all mice.

A divergence in IgG titers emerged at the four-week mark. While the control group (0 PFU) and the low-dose phage group (10^7^ PFU) showed only a moderate increase, the groups that received higher phage doses exhibited a pronounced and significant boost in antigen-specific antibody levels (Figure 3). The response was dose-dependent. The 10^8^ PFU group showed enhanced IgG, with values (0.368–0.376 OD) exceeding those of the control group (~0.31–0.33 OD). The 10^9^ PFU group demonstrated the most robust response, with peak IgG levels reaching approximately 0.48 OD, representing a nearly 1.5-fold increase over the control group’s peak.

### 3.3. PM16 Phage Activates Macrophages, Inducing Proinflammatory Cytokines and iNOS

Given that PM16 therapy enhances adaptive immunity in vivo, we investigated the underlying cellular mechanisms. We hypothesized that the phage primes innate immunity, with macrophages as a primary target. We therefore analyzed the direct transcriptional response of primary murine bone marrow-derived macrophages to PM16 (10^9^ PFU/mL) exposure in the absence of bacteria.

**Figure 4:**
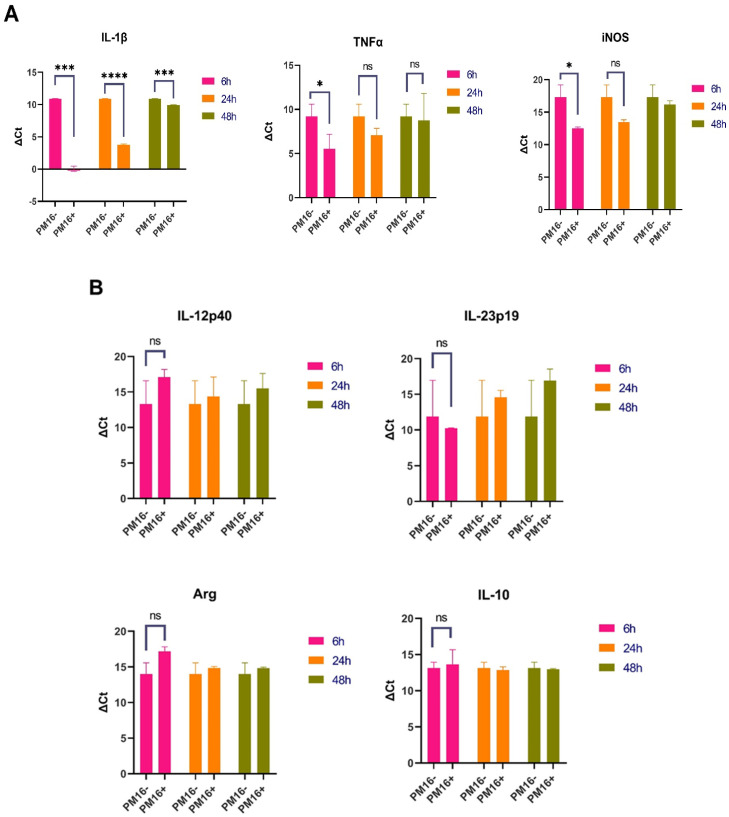

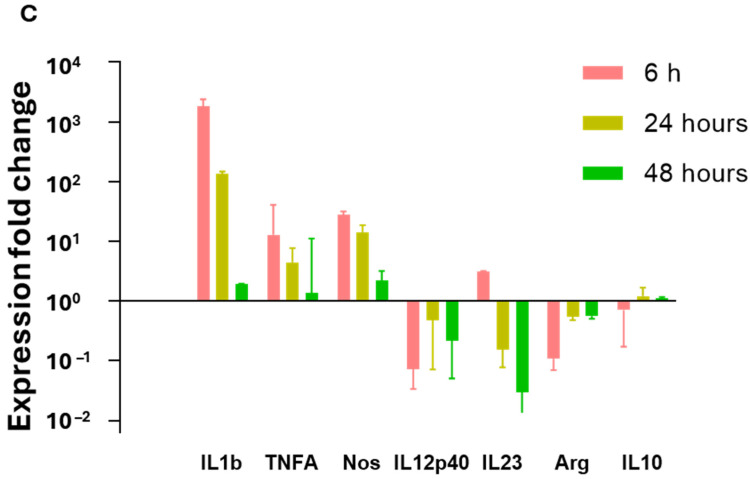
PM16 phage directly induces a specific and temporally regulated proinflammatory program in macrophages. Primary murine bone marrow-derived macrophages were stimulated with PM16 (10^9^ PFU/mL). Gene expression of key markers was analyzed by RT-qPCR at 6, 24, and 48 h post-stimulation and is presented as fold change relative to GM-CSF-stimulated controls. The plots represent the values of delta Ct between the gene of interest and the housekeeping gene. (**A**) PM16 induced a robust, early proinflammatory response. At 6 h, significant upregulation of IL-1β (>146-fold), TNFα (~17-fold), and iNOS (24–31-fold) was observed. By 48 h, cytokine expression subsided, while iNOS remained elevated. Data are presented as mean ± SEM from 3 independent experiments. Statistical significance was determined by Welch’s *t* test; * *p* < 0.05, *** *p* < 0.001, **** *p* < 0.0001 and “ns” for non-significant vs. control at the respective time point. (**B**) The PM16-induced signature was specific. Expression of IL-12p40, IL-23p19, Arginase-1 (Arg), and IL-10 remained at baseline levels (approximately 1-fold change) at both time points, indicating clear polarization toward a classical (M1) activation state without induction of alternative (M2) or other inflammatory markers. (**C**) The fold change plot calculated on delta-delta Ct and demonstrating changes in the expression level of the genes compared to the control expression level.

PM16 directly induced a robust proinflammatory gene expression profile in macrophages, consistent with classical M1 polarization (Figure 4A). Six h post-stimulation, the most pronounced upregulation was observed in the gene encoding IL-1β, which increased over 146-fold relative to the GM-CSF-stimulated controls (*p* < 0.001). Expression of TNFα was also significantly elevated, showing an approximate 17-fold increase (*p* < 0.05). Furthermore, we observed the substantial upregulation of iNOS, with expression levels increased 24- to 31-fold (*p* < 0.05). This shift suggests that PM16 not only triggers inflammatory signaling but also equips macrophages with machinery for enhanced direct bacterial killing.

This proinflammatory signature was specific. The expression of IL-12p40 was negligible, and IL-23p19 was uninduced (Figure 4B). Markers associated with alternative (M2) activation, Arginase-1 (Arg), and IL-10 remained at baseline levels. The strong induction of IL-1β, TNFα, and iNOS without a concomitant rise in Arg or IL-10 defines a macrophage activation state skewed toward host defense.

The macrophage response to PM16 was temporally regulated. By 48 h post-stimulation, the cytokine landscape had evolved. The upregulation of IL-1β had subsided to a modest 2-fold increase, whereas TNFα expression became more variable. In contrast, iNOS expression remained elevated above the baseline (2- to 3-fold), suggesting a sustained potential for antimicrobial activity (Figure 4A). The absence of IL-23p19, Arg, and IL-10 expressions was maintained (Figure 4B).

### 3.4. PM16 Enhances the Bactericidal Activity of Macrophages Against P. mirabilis

**Figure 5:**
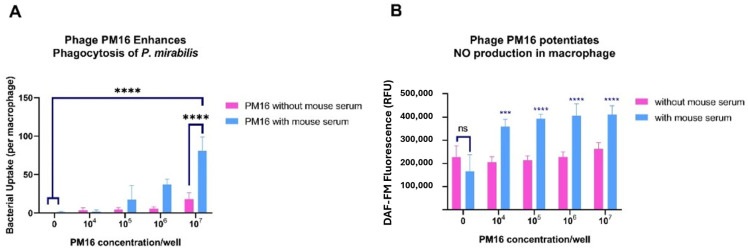
PM16 enhances bacterial uptake and NO production in macrophages. (**A**) Bacterial internalization by macrophages. Primary murine bone marrow-derived macrophages were pretreated with the indicated doses of PM16 for 30 min and then infected with *P. mirabilis* (10^7^ CFU/well) in the presence or absence of normal mouse serum. Intracellular bacteria were enumerated by confocal microscopy 5 h post-infection. Data are presented as the median number of bacteria per macrophage from three independent experiments. Statistical analysis was performed by two-way ANOVA with Sidak’s multiple comparisons test; **** *p* < 0.0001 and “ns”—non significant compared to the no-phage control. (**B**) NO production in macrophages. Macrophages were treated with the indicated doses of PM16 for 5 h in the presence or absence of serum, and intracellular NO was measured using DCF-DA fluorescence. Data are presented as fluorescence intensity relative to GM-CSF-only controls from three independent experiments. Statistical analysis was performed by two-way ANOVA with Sidak’s multiple comparisons test; *** *p* ≤ 0.0003 compared to the control group.

Taking into consideration that PM16 drives macrophages toward a proinflammatory state, we next assessed whether this activation enhances their ability to internalize and kill *P. mirabilis*. Using a combination of microscopy, fluorescence assays, and bacteriological culture, we evaluated bacterial uptake, NO production, and intracellular killing. Preincubation with PM16 significantly increased bacterial internalization by macrophages (Figure 5A). In the presence of normal mouse serum, the median bacterial count per macrophage rose with the increasing phage doses. At the highest phage concentration (10^7^ PFU/well), the median bacterial load reached 64–100 bacteria per cell compared to 0–2 bacteria per cell in the no-phage control (*p* < 0.0001).

Next, we measured NO production, a key bactericidal mechanism of activated macrophages. PM16 treatment, in the presence of murine serum, triggered an increase in NO production (Figure 4B) in comparison with the control group stimulated with GM-CSF only (*p* ≤ 0.0003).

**Figure 6:**
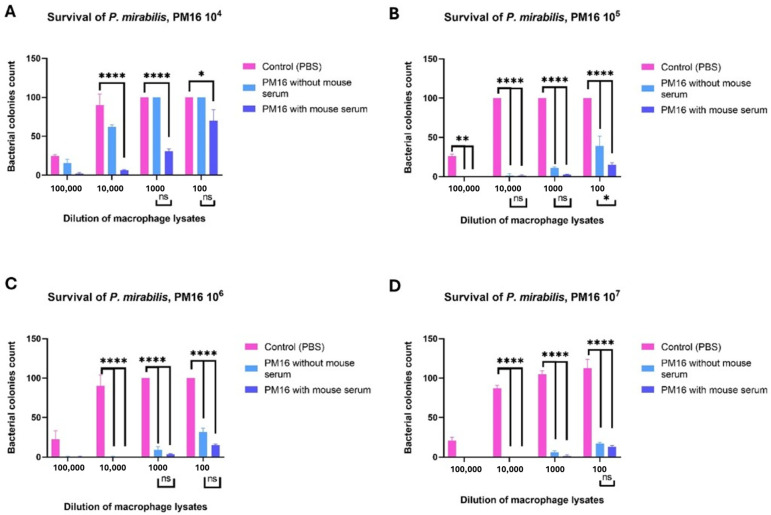
PM16 treatment enhances intracellular killing of *P. mirabilis* by macrophages in a dose-dependent manner. Primary murine bone marrow-derived macrophages were infected with *P. mirabilis* (10^7^ CFU/well) for 5 h and then treated with the indicated doses of PM16 with or without mouse serum. Twenty-four h post-treatment, macrophages were lysed, and the lysates were plated at the indicated dilutions to enumerate viable intracellular bacteria via colony counting. Data are representative of three independent experiments. (**A**) Macrophages treated with PM16 in concentration 10^4^ PFU/well, (**B**) Macrophages treated with PM16 in concentration 10^5^ PFU/well, (**C**) Macrophages treated with PM16 in concentration 10^6^ PFU/well, (**D**) Macrophages treated with PM16 in concentration 10^7^ PFU/well. Statistical analysis was performed by two-way ANOVA with Sidak’s multiple comparisons test. * *p* < 0.05, ** *p* < 0.001, **** *p* < 0.0001 and “ns”—non significant.

To determine the net effect on bacterial viability, we performed a survival assay by lysing macrophages 24 h post-treatment with PM16 and enumerating viable bacteria (Figure 6). PM16 treatment resulted in a dose-dependent reduction in recoverable colonies. At 10^5^ PFU/well, colony counts at the 1:100,000 dilution decreased from ~25 in controls to 0–3 in PM16-treated groups. At 10^7^ PFU/well, the bactericidal effect was nearly complete, with minimal colony formation even at lower dilutions, while controls showed confluent growth (recorded as 100+ colonies). No significant difference was detected between “without serum” and “with serum” groups.

## 4. Discussion

In this study, we present evidence that the *Proteus* phage PM16 acts not only as an antibacterial agent but also as a potent modulator of the immune response, enhancing the development of long-term, specific immunity against its bacterial host, *P. mirabilis*. Our in vivo results clearly indicated a dose-dependent protective effect of PM16 during both the first and second infections. In addition, the consequences of the protective effect of a single PM16 treatment were observed when mice were injected with *P. mirabilis* a month after treatment. This protective immunity was directly correlated with elevated levels of pathogen-specific IgG, indicating the successful induction of a robust adaptive immune response. Crucially, since we have previously shown that PM16 alone is unable to induce a long-term anti-phage antibody response [18], the observed enhancement cannot be attributed to the immunogenicity of the phage virions themselves. This finding contradicts the hypothesis that PM16 functions as a classic antigenic adjuvant.

Instead, we propose a mechanism centered on the priming of innate immunity. Our in vitro experiments established that PM16 is a direct and potent activator of macro-phages, driving a specific transcriptional program, which is consistent with classical M1 polarization. The potent induction of proinflammatory cytokines (TNFα, IL-1β) likely occurs through the recognition of phage nucleic acids by intracellular pattern recognition receptors, such as TLR9, a common pathway for immunostimulatory phages [18]. This activation is characterized by the robust, early upregulation of these key cytokines and a sustained induction of iNOS. More importantly, we demonstrated that this transcriptional shift is functionally significant: PM16-treated macrophages exhibit a dramatic increase in both bacterial uptake and intracellular killing. This aligns with the established concept of phage-mediated opsonization [15,21,22], where phages coating bacteria enhance their recognition and phagocytosis by immune cells. The potent, dose-dependent reduction in recoverable bacterial colonies in our survival assay confirms that the increased phagocytosis culminates in enhanced bacterial clearance, with nitric oxide being a key mediator of this bactericidal effect.

It should be mentioned that our in vitro findings revealed that the presence of normal mouse serum significantly amplified PM16-induced nitric oxide production in macrophages, even in the absence of bacteria. This suggests that serum components actively potentiate the phage’s immunostimulatory capacity. We propose that soluble serum factors, such as natural antibodies, or soluble pattern recognition receptors can bind to phage particles, forming complexes that more effectively engage macrophage surface receptors and/or enhance intracellular signaling. This serum-dependent synergy highlights the importance of considering the physiological context of phage–immune interactions, where blood and tissue fluids may critically modulate the immunomodulatory profile of therapeutic phages. This hypothesis was first proposed by Górski at al. [13], and future studies should be focused on specific serum components that can interact with bacteriophages.

Our observation that PM16 induces a strong proinflammatory cytokine profile stands in contrast to studies of other phages, such as T4, which have demonstrated anti-inflammatory properties, including the inhibition of NF-κB and reduction in reactive oxygen species (ROS) in immune cells [23]. This highlights the critical concept that phage–immune interactions are phage-specific. The immune net outcome—whether proinflammatory, anti-inflammatory, or neutral—depends on the specific phage, its structural components, and its interaction with host receptors [15,24]. The robust M1 polarization induced by PM16 suggests that it engages different signaling pathways than other phages, which elicited anti-inflammatory markers like the IL-1 receptor antagonist (IL1RN), or suppresses LPS-driven inflammation [24,25,26]. This specificity underscores the importance of a detailed characterization of the immunomodulatory “fingerprint” of each therapeutic phage candidate.

Finally, the translational implication of this primed state is significant. By enhancing phagocytosis and intracellular killing, PM16 not only aids in direct bacterial clearance but also promotes more efficient processing of bacterial antigens. This process is essential for linking innate detection to adaptive immunity. The observed long-term protective memory likely stems from this optimized antigen presentation, a hypothesis supported by models of phage–immune synergy [27], where effective pathogen handling by primed innate cells is a prerequisite for durable adaptive responses. Future work should directly trace the fate of bacterial antigens from PM16-treated mice to B and T-cell activation to confirm this proposed link.

In conclusion, this study repositions the PM16 podophage from a simple antibacterial agent to an active partner and primer of the immune system. Its specific capacity to skew macrophages toward a bactericidal, proinflammatory phenotype via innate receptor recognition creates a local microenvironment that ensures effective pathogen clearance and orchestrates a superior adaptive immune response, resulting in durable protection against reinfection.

## Figures and Tables

**Table 1:** Sequences of primers used for RT-PCR analysis.

Cytokine	Primer Set	Tm
Murine IL-1b	Forward: 5′ TGCCACCTTTTGACAGTGATG 3′ Reverse: 5′ TGATGTGCTGCTGCGAGATT 3′	60
Murine IL12p40	Forward: 5′ GGAGGGGTGTAACCAGAAAGG 3′ Reverse: 5′ TAGCGATCCTGAGCTTGCAC 3′	59
Murine IL23	Forward: 5′ ACCAGCGGGACATATGAATCT 3′ Reverse: 5′ AGACCTTGGCGGATCCTTTG 3′	59
Murine inducible NO-synthase	Forward: 5′ GCTCCCTATCTTGAAGCCCC 3′ Reverse: 5′ TGGAAGCCACTGACACTTCG 3′	58
Murine IL10	Forward: 5′ GTAGAAGTGATGCCCCAGGC 3′ Reverse: 5′ GACACCTTGGTCTTGGAGCTTATT 3′	60
Murine Arginase 1	Forward: 5′ TTTCTCAAAAGGACAGCCTCG 3′ Reverse: 5′ CAGACCGTGGGTTCTTCACA 3′	58
Murine IL4	Forward: 5′ TCACAGCAACGAAGAACACCA 3′ Reverse: 5′ CAGGCATCGAAAAGCCCGAA 3′	58

## Data Availability

The original contributions presented in this study are included in the article. Further inquiries can be directed to the corresponding authors.

## References

[1] Schaffer J.N., Pearson M.M. (2015). *Proteus mirabilis* and Urinary Tract Infections. Microbiol. Spectr..

[2] Jacobsen S.M., Stickler D.J. (2008). Complicated Catheter-Associated Urinary Tract Infections Due to *Escherichia coli* and *Proteus mirabilis*. Clin. Microbiol. Rev..

[3] Stock I. (2003). Natural Antibiotic Susceptibility of *Proteus* spp., with Special Reference to *P. mirabilis* and *P. penneri* Strains. J. Chemother..

[4] Hafiz T.A., Alghamdi G.S., Alkudmani Z.S., Alyami A.S., AlMazyed A., Alhumaidan O.S., Mubaraki M.A., Alotaibi F.E. (2024). Multidrug-Resistant *Proteus mirabilis* Infections and Clinical Outcome at Tertiary Hospital in Riyadh, Saudi Arabia. Infect. Drug Resist..

[5] Kynshi M.A.L., Kharkamni E., Borah V.V. (2025). *Proteus mirabilis*: Insights into biofilm formation, virulence mechanisms, and novel therapeutic strategies. Microbe.

[6] Wang Y., Yu Y. (2025). Phage therapy as a revitalized weapon for treating clinical diseases. Microbiome Res. Rep..

[7] Moon K., Coxon C., Ardal C., Botgros R., Djebara S., Durno L., Fiore C.R., Perrin J.-B., Dixon D.M., Cavaleri M. (2025). Considerations and Perspectives on Phage Therapy from the Transatlantic Taskforce on Antimicrobial Resistance. Nat. Commun..

[8] Thompson T., Kilders V., Widmar N., Ebner P. (2024). Consumer Acceptance of Bacteriophage Technology for Microbial Control. Sci. Rep..

[9] Youssef R.A., Sakr M., Shebl R.I., Aboshanab K.M. (2026). Recent insights on challenges encountered with phage therapy against gastrointestinal-associated infections. Gut Pathog..

[10] Levin B.R., Bull J.J. (2004). Population and Evolutionary Dynamics of Phage Therapy. Nat. Rev. Microbiol..

[11] Marchi J., Zborowsky S., Debarbieux L., Weitz J.S. (2023). The Dynamic Interplay of Bacteriophage, Bacteria and the Mammalian Host during Phage Therapy. iScience.

[12] Weissfuss C., Li J., Behrendt U., Hoffmann K., Bürkle M., Tan C., Krishnamoorthy G., Korf I., Rohde C., Gaborieau B. (2025). Adjunctive phage therapy improves antibiotic treatment of ventilator-associated-pneumonia with *Pseudomonas aeruginosa*. Nat. Commun..

[13] Górski A., Dąbrowska K., Międzybrodzki R., Weber-Dąbrowska B., Łusiak-Szelachowska M., Jończyk-Matysiak E., Borysowski J. (2017). Phages and immunomodulation. Future Microbiol..

[14] Górski A., Międzybrodzki R., Łobocka L., Głowacka-Rutkowska A., Bednarek A., Borysowski J., Jończyk-Matysiak E., Łusiak-Szelachowska M., Weber-Dąbrowska B., Bagińska N. (2018). Phage Therapy: What Have We Learned?. Viruses.

[15] Van Belleghem J., Dąbrowska K., Vaneechoutte M., Barr J., Bollyky P. (2018). Interactions between Bacteriophage, Bacteria, and the Mammalian Immune System. Viruses.

[16] Jończyk-Matysiak E., Weber-Dąbrowska B., Owczarek B., Międzybrodzki R., Łusi-ak-Szelachowska M., Łodej N., Górski A. (2017). Phage-Phagocyte Interactions and Their Implications for Phage Application as Therapeutics. Viruses.

[17] Podlacha M., Grabowski Ł., Kosznik-Kawśnicka K., Zdrojewska K., Stasiłojć M., Węgrzyn G., Węgrzyn A. (2021). Interactions of Bacteriophages with Animal and Human Organisms—Safety Issues in the Light of Phage Therapy. Int. J. Mol. Sci..

[18] Chechushkov A., Kozlova Y., Baykov I., Morozova V., Kravchuk B., Ushakova T., Bardasheva A., Zelentsova E., Allaf L.A., Tikunov A. (2021). Influence of Caudovirales Phages on Humoral Immunity in Mice. Viruses.

[19] Morozova V., Kozlova Y., Shedko E., Kurilshikov A., Babkin I., Tupikin A., Yunusova A., Chernonosov A., Baykov I., Kondratov I. (2016). Lytic Bacteriophage PM16 Specific for *Proteus mirabilis*: A Novel Member of the Genus Phikmvvirus. Arch. Virol..

[20] Sambrook J., Russell D.W. (2001). Molecular Cloning: A Laboratory Manual.

[21] Popescu M., Van Belleghem J.D., Khosravi A., Bollyky P.L. (2021). Bacteriophages and the Immune System. Annu. Rev. Virol..

[22] Jończyk-Matysiak E., Łusiak-Szelachowska M., Kłak M., Bubak B., Międzybrodzki R., Weber-Dąbrowska B., Żaczek M., Fortuna W., Rogóż P., Letkiewicz S. (2015). The Effect of Bacteriophage Preparations on Intracellular Killing of Bacteria by Phagocytes. J. Immunol. Res..

[23] Miernikiewicz P., Kłopot A., Soluch R., Szkuta P., Kęska W., Hodyra-Stefaniak K., Konopka A., Nowak M., Lecion D., Kaźmierczak Z. (2016). T4 Phage Tail Adhesin Gp12 Counteracts LPS-Induced Inflammation In Vivo—Pub-Med. Front. Microbiol..

[24] Górski A., Międzybrodzki R., Borysowski J., Dąbrowska K., Wierzbicki P., Ohams M., Korczak-Kowalska G., Olszowska-Zaremba N., Łusiak-Szelachowska M., Kłak M. (2012). Phage as a Modulator of Immune Responses: Practical Implications for Phage Therapy. Adv. Virus Res..

[25] Międzybrodzki R., Borysowski J., Kłak M., Jończyk-Matysiak E., Obmińska-Mrukowicz B., Suszko-Pawłowska A., Bubak B., Weber-Dąbrowska B., Górski A. (2017). In Vivo Studies on the Influence of Bacteriophage Preparations on the Autoimmune Inflammatory Process. Biomed. Res. Int..

[26] Miedzybrodzki R., Switala-Jelen K., Fortuna W., Weber-Dabrowska B., Przerwa A., Lusiak-Szelachowska M., Dabrowska K., Kurzepa A., Boratynski J., Syper D. (2008). Bacteriophage Preparation Inhibition of Reactive Oxygen Species Generation by Endotoxin-Stimulated Polymorphonuclear Leukocytes—PubMed. Virus Res..

[27] Roach D.R., Leung C.Y., Henry M., Morello E., Singh D., Di Santo J.P., Weitz J.S., Debarbieux L. (2017). Synergy between the Host Immune System and Bacteriophage Is Essential for Successful Phage Therapy against an Acute Respiratory Pathogen. Cell Host Microbe.

